# Supplementary material for: In vivo generation of DNA sequence diversity for cellular barcoding
Source: Nucleic Acids Res. 2014 Jul 10;42(16):e127. doi: 10.1093/nar/gku604 (PMC4176322; doi:10.1093/nar/gku604)
Supplement: SUPPLEMENTARY DATA [file supp_42_16_e127__index.html]

In vivo generation of DNA sequence diversity for cellular barcoding — SUPPLEMENTARY DATA 

# *In vivo* generation of DNA sequence diversity for cellular barcoding

## SUPPLEMENTARY DATA

**Files in this Data Supplement:**

- Supplementary Data
