# Supplementary material for: In vivo generation of DNA sequence diversity for cellular barcoding
Source: Nucleic Acids Res. 2014 Jul 10;42(16):e127. doi: 10.1093/nar/gku604 (PMC4176322; doi:10.1093/nar/gku604)
Supplement: SUPPLEMENTARY DATA [file supp_gku604_nar-01090-met-k-2014-File008.zip › NAR_RCI_SUPP_ip1.docx]

***In vivo* generation of DNA sequence diversity for cellular barcoding**

**Sequences of relevant genetic elements**

**>5BC_Cassette (synthesized by IDT)**

gctttacctcgcactgcccagagtgacatgtttgcgaacctcatcactcgttgcggcaat

actttcgtgccaatccggtacgtggtgtatgcaagggaaattaacggacggcctcaattc

ctgcaaggtaacgcaccgcggcccaagagccattaagcgatataatcgcacatctggcca

acccgccacgtaccggattggcacgcttgcagagaatcctgggctcttcaaccaacagtg

acgggggcttattaggaggattttgatacggacgcgcaaccgtcgtcaggcagctttgaa

gcccgtctctccggatgccaagcttgttgtgccaatccggtacgtggcggccgagttcgc

tcacctttttgaatcctcggacgccataactaacagcccgtttatggaagagtatgcact

gcctaagggcggagggccaagagtcctccaggtaccggattggcactggcctacgtgccc

caattaccacattaaagatatctgcactggcgtcccctcttctcgaggacgagggtaaaa

aagcgccattgcactaggacttaccgcggagactgcctctgctggcctacgtgccaatcc

ggtacgtggcgttgcatgtattgcagcctcagggacgtcagtggatcatgaaggtagagc

atgcgtcctctgctgttaaaatctgagttctggacaaactaccaattggccacgtaccgg

attggcacgaaagtattgccggacagcatcttccttgcctcaacatgtcgaacacagtgg

cacgatgcatgagtct

**>BCextension (synthesized by GeneWiz)**

CGCGCTAGCGGCAATACTTTCGTGCCAATCCGGTACGTGGGATATAGGATTGATTTTTCT

TCACTGTACGCCGGAGAACATAAGAAGCGTCATAACCCTATCAGTCCCAAGAATAAGTCC

CAGTATTAACTGTTTCAGCACCACGTACCGGATTGGCACGCTTGCAGAGAAATAGGGAAG

GCTCATAGCGTTCGAAGTAGTTCCCAACCAGAACACTCCTGGTGAGTCGCTGGAAGCCAT

CTATAGAAAGGACGACGCTCGGGCCGGTGTAGCCAAGCTTGTTGTGCCAATCCGGTACGT

GGACAACGTCAACGTTAATCTCGTGCATCAGATCCTAGTTGGTTGAGAACAGAGCGCGAC

GTGGGGCTGGGAGCCGATACCCGTAAGTAGGAGAGGGCAGCCCCAGGTACCGGATTGGCA

CTGGCCTACGTGCGCCATCGCACCATTAAACATACCCGGATCGATATTTAGGTGTGCGGG

GCTCCTGCATCAATTAATACATCGTTCGAACCAGACCCGTCCATTCATCTCGCCTGCTGG

CCTACGTGCCAATCCGGTACGTGGGCGATTAATCAGCATCCAAGGTTGCACACCGGTTTA

ATCGTGGGACATCGTCAAGCGTCAATATCCTAAAGACCTGCGAGGTTTAGGCATCAGTGC

GGGGCCACGTACCGGATTGGCACGAAAGTATTGCCACCTCGAAAGTGACGATTGTTATTG

ATCGGTTTGCACCCCGACCAGCCAGGCATCTCATCTCCACGGGGATCGAGGGCATCAATC

ACATTTGACGGATCCGCG

**>Rci sequence (from NCBI Reference Sequence: NC_013120.1 REGION: complement 72912…74066)**

atgccgtctccacgcatccgtaaaatgtccctgtcacgcgcactggataagtacctgaaa

acagtttctgttcacaagaaagggcatcaacaggagttttaccggagcaatgttatcaag

cgatatcccattgctcttcggaatatggacgaaataacaaccgttgatattgctacatac

agagacgttcgtttagcagaaataaacccccgaacgggtaaagccattacaggtaatact

gtacgtcttgaactcgcccttctgtcatctctgttcaatattgctcgtgttgaatgggga

acctgtcgtactaacccggttgaactggttcgcaagccgaaagtatcctccggacgagat

cgccggctaacgtcttcagaagaacgtcgcctttctcgctatttccgcgaaaaaaatctg

atgttgtatgtcattttccatcttgcccttgaaacagccatgcggcagggcgaaatactg

gccttacgttgggagcacattgatttgcgccacggtgtggctcatttacctgaaaccaaa

aacggtcactcacgggatgttcctctgtccagacgtgcccgtaactttcttcaaatgatg

cccgttaatctccacggcaatgtttttgattacaccgcatccggctttaaaaatgcctgg

agaatagccacacaacgacttcgcatcgaggacctgcattttcacgatctacggcatgaa

gcaataagccgcttcttcgaactgggtagcctgaatgtaatggagattgctgcaatatca

ggacatcgttccatgaatatgctgaaacggtatactcatcttcgtgcatggcaactggtc

agtaagcttgatgcccgccggcggcagacacaaaaagtggcagcatggtttgtgccgtat

cctgcccatatcacgactatcgatgaagaaaatgggcagaaagcgcatcgtattgagatc

ggtgattttgataaccttcacgtcactgccacaacaaaagaggaagcagttcaccgcgcc

agtgaggttttgttgcgtacactggccattgcagcacagaaaggcgaacgtgtcccatct

cccggagcgttacctgttaacgaccctgactacattatgatttgccctctgaacccgggc

agcacaccgctgtaa

**Plasmid maps provided:**

All plasmids and associated maps are included here, and on Benchling.com: <https://benchling.com/ipeikon/ipeikon_published/>

- IDP190: 5BC Cassette
- IDP205: T7->Rci; 5BC Cassette
- DIG35: pKat->Rci; 5BC Cassette
- BCextension: 6 fragment BC extension
- DIG70: T7->Rci; 11BC Cassette
- DIG71: pKat->Rci; 11BC Cassette

**Description of Supplementary Files:**

- **crecode.m :** Simulates Cre recombination on a cassette where fragments are separated by two lox sites in opposing orientation
- **rcicode.m :** Simulates Rci recombination on a cassette where fragments are separated by one sfx site in alternating orientation
- **rcicode2.m :** Simulates Rci recombination on a cassette where fragments are separated by two sfx sites in opposing orientation
- **randDNA.m :** Generates random DNA sequences for boostrapping SW alignment thresholds
- **procRCI.m :** Processes data from Sanger sequencing to reconstruct barcodes
- **procRCI_PB.m :** Processes data from PacBio sequencing to reconstruct barcodes

**Supplementary Notes**

**Supplementary Note 1**

Simulations (see Methods for details) suggested that the Cre cassettes are subject to considerable biases. Specifically, the ends of barcode fragments are favored for retention (Supplementary Figure S1a). This is explained by the simple observation that there are more combinations of lox sites that, when acted on by Cre, will result in the excision of middle fragments (Supplementary Figure S1b). These inherent biases severely limit the practical diversities that can be generated with Cre-based cassettes.

**Supplementary Figures**

**Supplementary Figure 1.** Biases of the Cre architecture **(A)** Simulated Cre recombination on 10,000 cassettes of length *n*=100 reveals extreme biases for retaining end fragments. **(B)** There are many more pairs of *lox* sites that can lead to the excision of more central fragments.

**Supplementary Figure 2.** 5BC Cassette stability. Sanger sequencing of the 5BC cassette after several generations of growth in bacterial cells shows no recombination of the cassette.

**Supplementary Figure 3**. T7 induced Rci expression results in single recombination events. All of the reconstructed sequences resulting from shuffling by induced expression of Rci from the T7 promoter can be explained by a single recombination event.

**Supplementary Figure 4.** 11BC Cassette stability**.** Sanger sequencing of the 11BC cassette after several generations of growth in bacterial cells shows no recombination of the cassette.

**Supplementary Figure 5.** Cassettes approach complete randomness as the number of recombination events increase. **(A)** Simulated cassettes subjected to 5, 10, or 15 random recombination events. The colormaps show the distribution of fragment occupancy at each position in the cassette. Colors are scaled from 0 to 25%. **(B)** The bias at each position was calculated as the number of times the original fragment appeared in its original position divided by the number of cassettes. The dotted black line indicates the expected occupancy of the original fragment at each position in a cassette with completely random occupancy.

**Sequencing Data Files:**

- 5BC_CCS_IDP205.fastq
  - No Rci expression (T7->Rci; 5BC Cassette)
- 5BC_CCS_DIG35.fastq
  - Rci expression (pKat->Rci; 5BC Cassette)
- 11BC_CCS_DIG71.fastq
  - Rci expression (pKat->Rci; 11BC Cassette)
