# Supplementary figures and images for: In vivo generation of DNA sequence diversity for cellular barcoding
Source: Nucleic Acids Res. 2014 Jul 10;42(16):e127. doi: 10.1093/nar/gku604 (PMC4176322; doi:10.1093/nar/gku604)

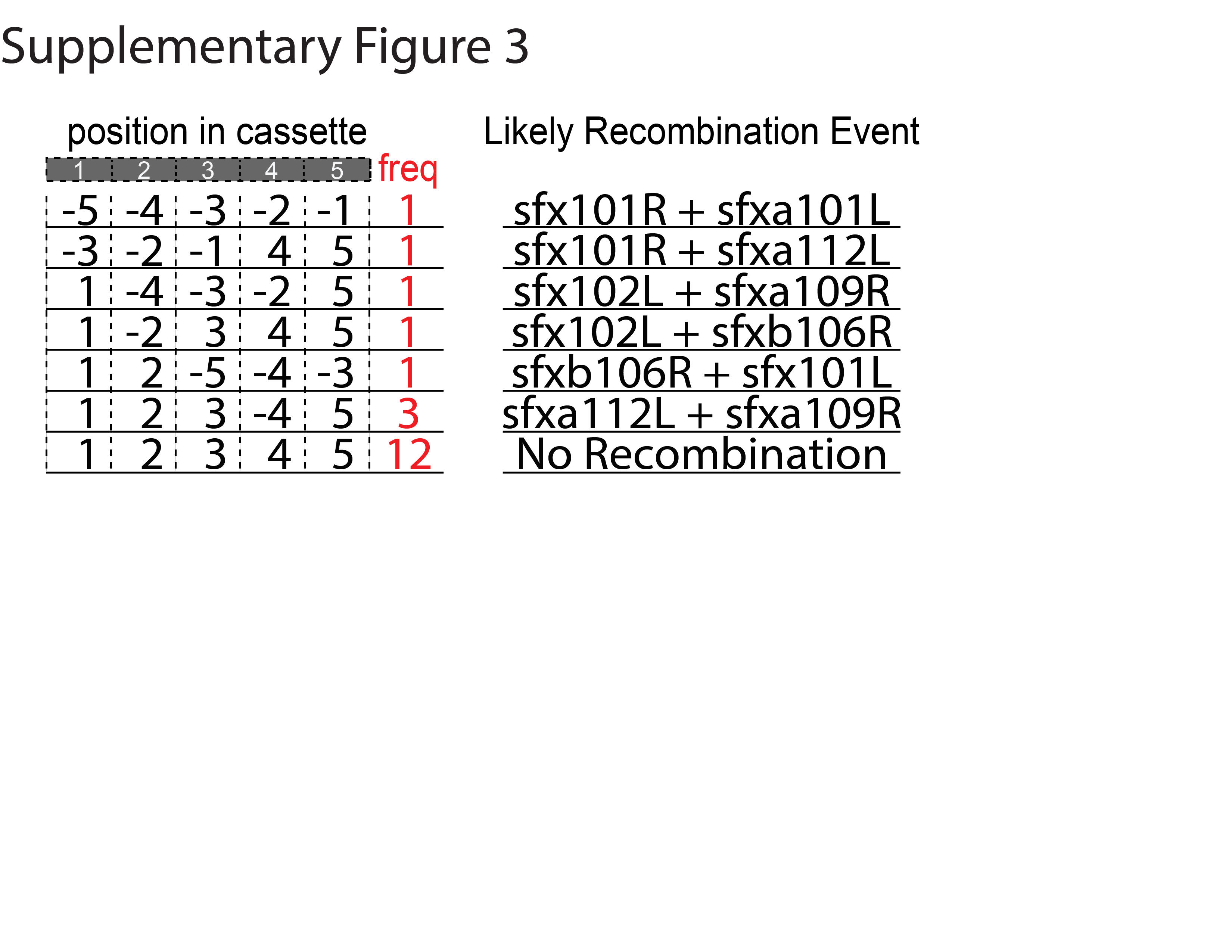

Supplement: SUPPLEMENTARY DATA [file supp_gku604_nar-01090-met-k-2014-File008.zip › SupplementaryALL_NAR_v1_rev1-03.png]

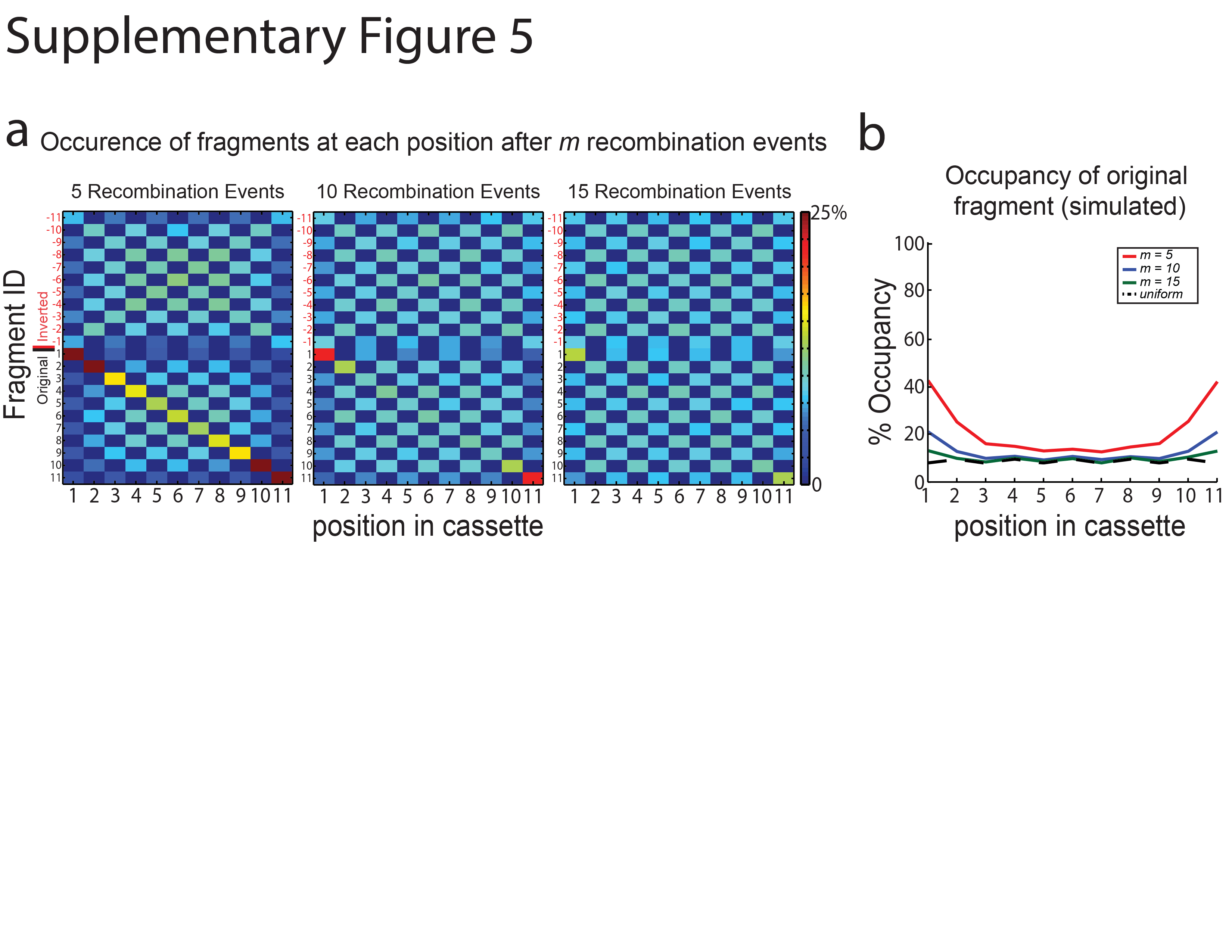

Supplement: SUPPLEMENTARY DATA [file supp_gku604_nar-01090-met-k-2014-File008.zip › SupplementaryALL_NAR_v1_rev1-05.png]

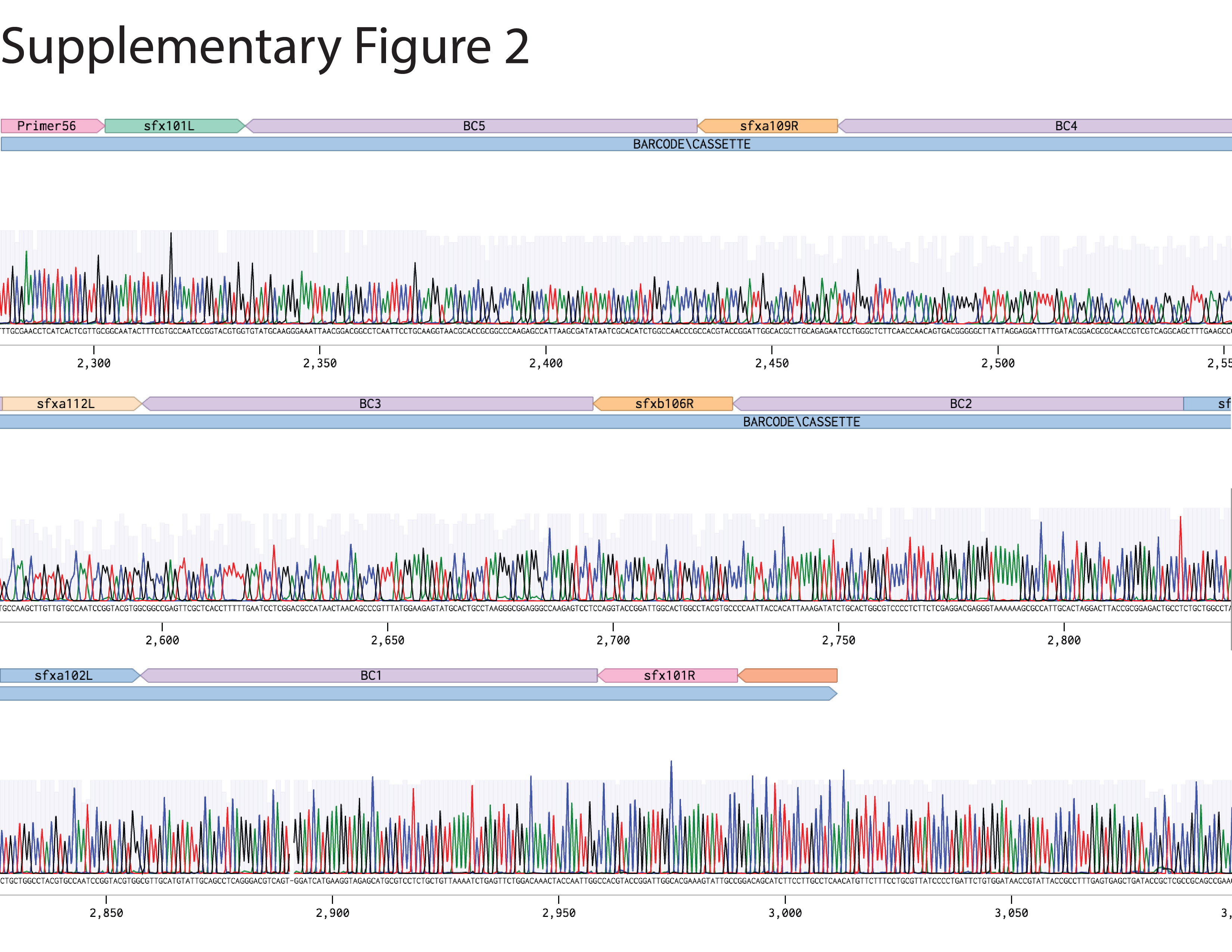

Supplement: SUPPLEMENTARY DATA [file supp_gku604_nar-01090-met-k-2014-File008.zip › SupplementaryALL_NAR_v1_rev1-02.png]

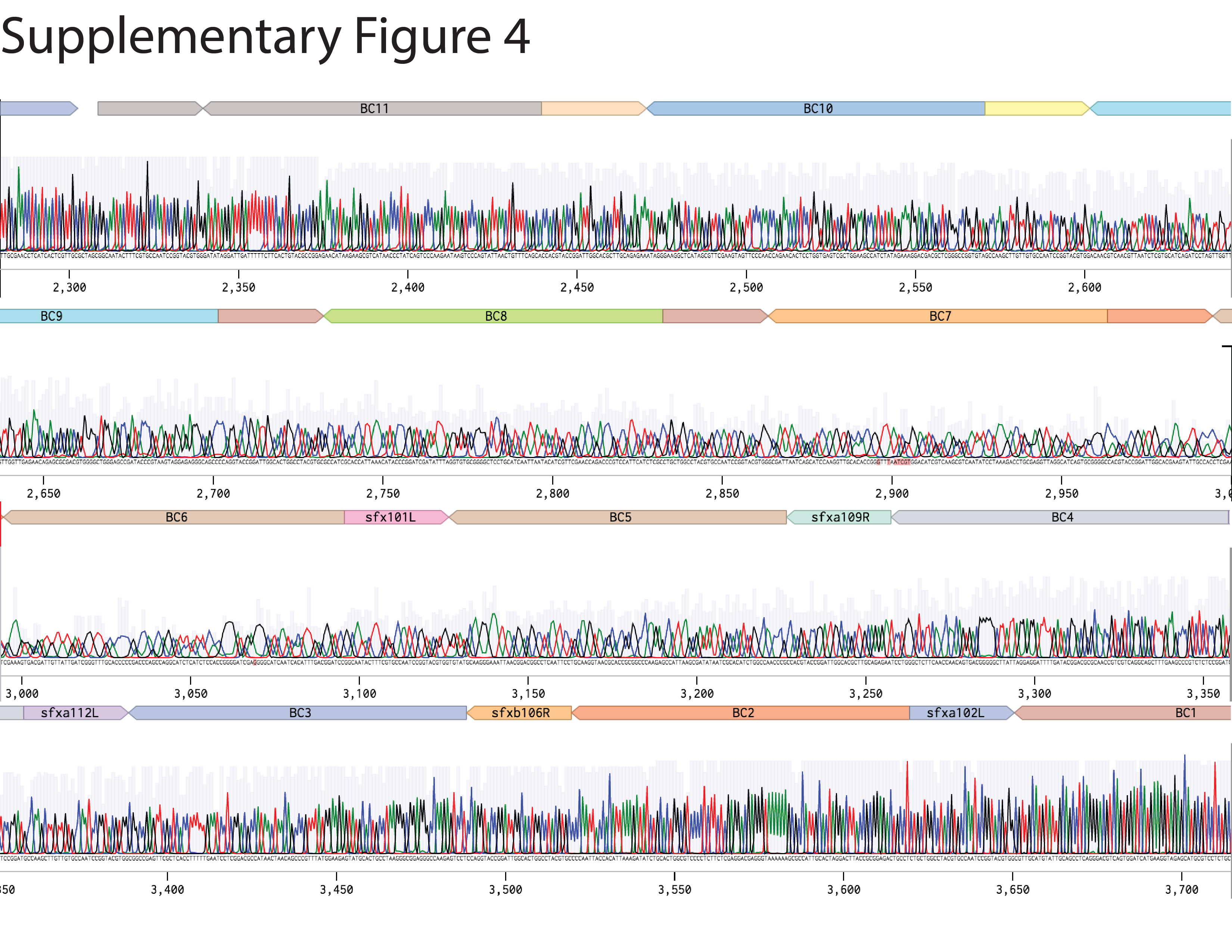

Supplement: SUPPLEMENTARY DATA [file supp_gku604_nar-01090-met-k-2014-File008.zip › SupplementaryALL_NAR_v1_rev1-04.png]

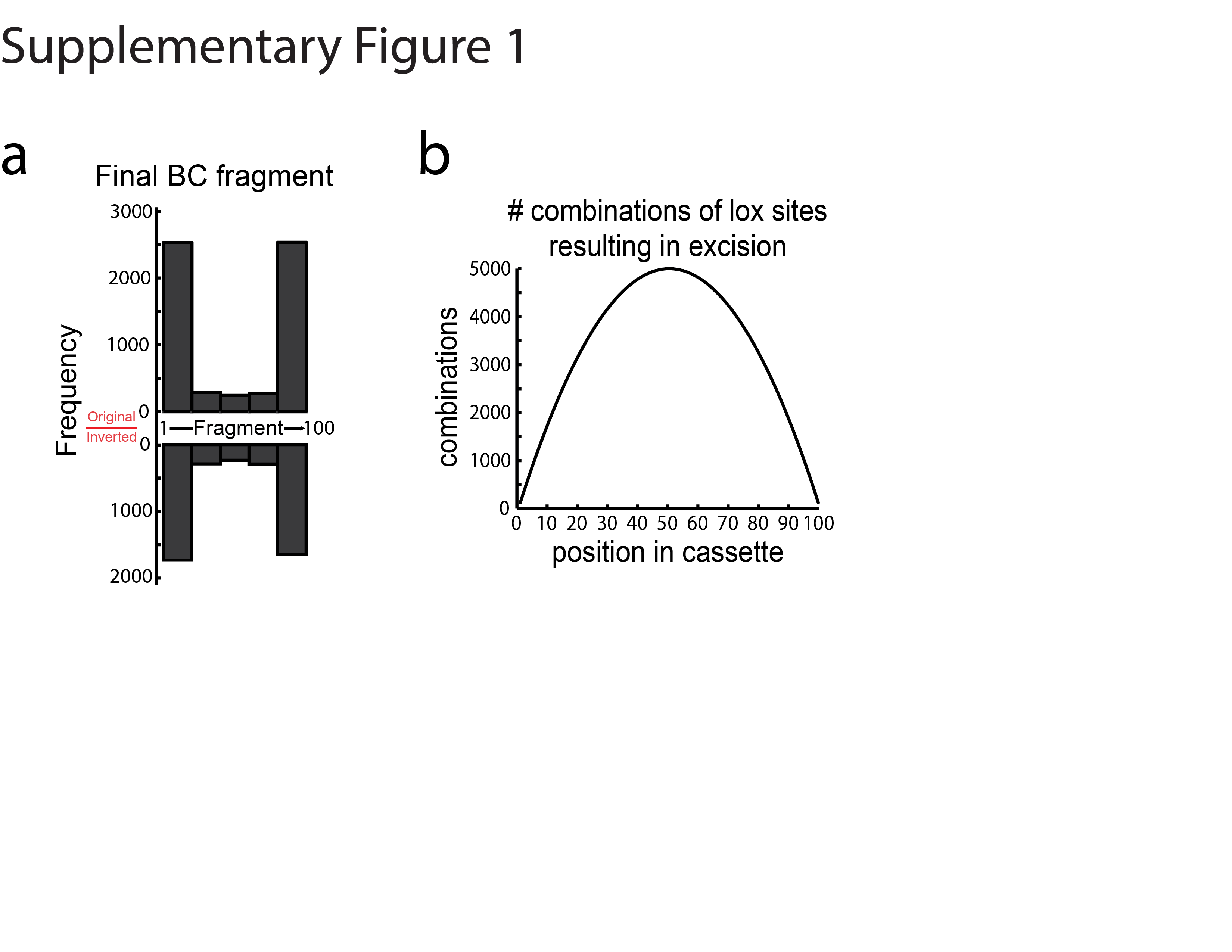

Supplement: SUPPLEMENTARY DATA [file supp_gku604_nar-01090-met-k-2014-File008.zip › SupplementaryALL_NAR_v1_rev1-01.png]
